# Patient Perspectives on Digital Interventions to Manage Heart Failure Medications: The VITAL-HF Pilot

**DOI:** 10.3390/jcm12144676

**Published:** 2023-07-14

**Authors:** Marc D. Samsky, Renee Leverty, James M. Gray, Alexandra Davis, Brett Fisher, Ashul Govil, Tom Stanis, Adam D. DeVore

**Affiliations:** 1 Section of Cardiovascular Medicine, Yale University School of Medicine, New Haven, CT 06520, USA; 2 The Duke Clinical Research Institute, Durham, NC 27710, USA; 3 Duke Heart Center, Duke University School of Medicine, Durham, NC 27710, USA; 4 Story Health, Cupertino, CA 95014, USAtom@storyhealth.ai (T.S.)

**Keywords:** guideline-directed medical therapy, digital health, virtual health, remote monitoring

## Abstract

Use of guideline-directed medical therapy (GDMT) for treatment of heart failure with reduced ejection fraction (HFrEF) remains unacceptably low. The purpose of this study was to determine whether a digital health tool can augment GDMT for patients with HFrEF. Participants ≥ 18 years old with symptomatic HFrEF (left ventricular ejection fraction ≤ 40%) and with access to a mobile phone with internet were included. Participants were given a blood pressure cuff, instructed in its use, and given regular symptom surveys via cell-phone web-link. Data were transmitted to the Story Health web-based platform, and automated alerts were triggered based on pre-specified vital sign and laboratory data. Health coaches assisted patients with medication education, pharmacy access, and lab access through text messages and phone calls. GDMT titration plans were individually created in the digital platform by local clinicians based on entry vitals and labs. Twelve participants enrolled and completed the study. The median age and LVEF were 52.5 years (IQR, 46.5–63.5) and 25% (IQR, 22.5–35.5), respectively. There were 10 GDMT initiations, 52 up-titrations, and 13 down-titrations. Five participants engaged in focus-group interviews following study completion to understand first-hand perspectives regarding the use of digital tools to manage GDMT. Participants expressed comfort knowing that there were clinicians regularly reviewing their data. This alleviated concerns of uncertainty in daily living, led to an increased feeling of security, and empowered patients to understand decision-making regarding GDMT. Frequent medication changes, and the associated financial impact, were common concerns. Remote titration of GDMT for HFrEF is feasible and appears to be a patient-centered approach to care.

## 1. Introduction

Heart failure is a major public health concern affecting more than 6 million Americans. The care of patients with heart failure places a significant burden on the United States healthcare system in terms of morbidity, mortality, and cost. In 2012, there were nearly 2 million clinic visits, 500,000 emergency visits, and over 1 million hospital discharges (≈25% of discharged patients are readmitted within 30 days) for heart failure-related care, with an associated total cost of USD 30 billion. Projections estimate USD 70 billion in total heart failure related spending by 2030 [1].

Large, randomized clinical trials have repeatedly demonstrated the benefits of several classes of medications for the treatment of patients with heart failure with a reduced ejection fraction (HFrEF). Additionally, imputed analyses of guideline-directed medical therapy (GDMT) for eligible patients with HFrEF have repeatedly demonstrated reduced morbidity, mortality, and costs [2,3]. As a result, international cardiovascular professional societies assign the highest recommendations to use these therapies in all eligible patients [4,5]. Despite these findings and recommendations, the use of guideline-directed medical therapy for patients with HFrEF remains low [6,7]. 

There are many potential reasons for under-dosing of GDMT. Clinical inertia, impracticality of frequent clinical visits, and patient factors have all been hypothesized to contribute [8,9]. Comprehensive disease management programs have been designed to provide targeted, specialized multidisciplinary care aimed at improving outcomes, via alleviating barriers to patient access, providing education, and promoting patient self-care. There has been heterogeneity in reported results of these programs [10,11,12,13,14,15]. 

In response to the defined role of heart failure disease management programs combined with the known benefits, and current underutilization of GDMT, The American College of Cardiology and American Heart Association have specifically identified understanding systematic approaches to HFrEF management, in particular GDMT initiation and optimization, as an area in need of further investigation [4]. Mobile, remote-based strategies to augment GDMT might provide a potentially elegant solution by reducing the resources required to manage a growing population of patients. To address this unmet need, the Virtual Care to Improve Heart Failure Outcomes (VITAL-HF) pilot was designed to determine if a digital health tool can augment GDMT use for patients with HFrEF.

## 2. Materials and Methods

### 2.1. Study Population

Participants were required to be ≥18 years-old, have HFrEF defined as a left ventricular ejection fraction ≤40%, documented within 12 months, with accompanying symptoms attributed to a decreased ejection fraction, and have access to a mobile phone with internet. A left ventricular ejection fraction of ≤40% was chosen based on international professional society practice guidelines for treatment of HFrEF [4,5,16]. All participants were also required to provide written consent. Participants were enrolled from a specialty heart failure clinic or before a heart failure-related hospital discharge, and were also required to receive follow up care at our institution. Patients were excluded from participation if they met any of the following criteria: received or were listed for cardiac transplantation, supported with or were planning to undergo placement of a durable left ventricular assist device, supported with intravenous inotropes, had end-stage kidney disease on renal replacement therapy, had a diagnosis of infiltrative cardiomyopathy, had pulmonary hypertension requiring specialized pharmacotherapy, had an arm circumference of >42 cm (size of an extra-large blood pressure cuff), were currently pregnant or breastfeeding, or would be unable to adhere to the protocol based on the discretion of the investigator. 

### 2.2. Study Design

The VITAL-HF pilot was designed as a single-arm feasibility study to determine whether a digital health therapeutic tool (Story Health, Cupertino, CA, USA) can augment GDMT for patients with ambulatory HFrEF outside of traditional clinic settings. After a minimum of 3 months in the study, participants were invited to a group-based interview to understand first-person accounts of perspectives while participating in VITAL-HF. 

### 2.3. Intervention and Study Procedures

**Figure 1:**
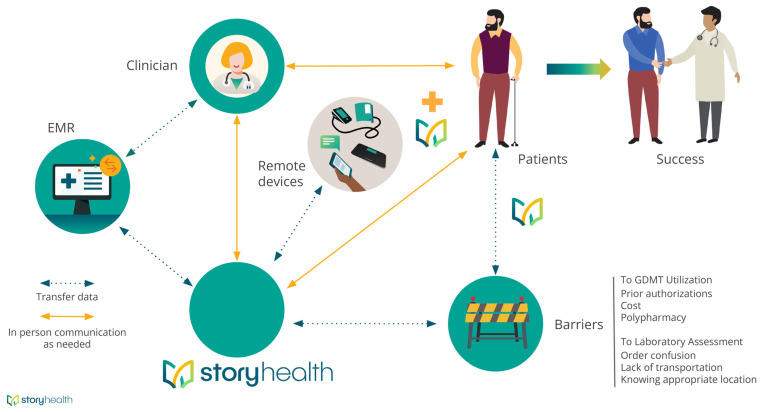
Illustration demonstrating how participants, clinicians, and Story Health interacted during the study. Dashed green lines represent flow of information, and solid lines represent ability for direct communication, as needed. The Story Health logo is depicted at various places in the schema to represent direct patient assistance as needed.

Following enrollment, a tailored HFrEF GDMT titration plan was individually created by study clinicians, with consideration given to baseline blood pressure (BP), heart rate, and serum chemistry profile [17]. Participants were provided with an appropriately sized home BP cuff and were trained for its proper use. Patients were instructed to measure their BP and heart rate, and to fill out a symptom log via cell-phone web-link. Measurements were transmitted in real-time to the Story Health (Cupertino, CA, USA) platform, and uploaded to the web-based portal for review. Automated alerts were triggered for clinician review, when appropriate, based on pre-specified thresholds of BP, heart rate, and reported symptoms. Alerts were reviewed, on average 3 or 4 times weekly. After 1 week of acceptable measurements, an automated titration alert was sent to the study team. If clinician-approved, a prescription was sent to a local pharmacy and, if necessary, laboratory orders were generated for serum potassium and renal function assessment following medication titration. Health coaches assisted patients with BP cuff use, symptom reporting, medication education, pharmacy access, and lab access both through text message and through phone calls. Following each medication titration, participants underwent a 3-day frequent vital-sign assessment and were instructed to use their BP cuff at least twice daily (Figure 1). Prespecified emergent alerts, including blood pressure readings or serum potassium levels, were required to be adressed in real-time. Clinicians were alerted by the Story Health platform. Prespecified critical alerts included symptoms associated with systolic blood pressure >200 mmHg or diastolic blood pressure >130 mmHg. Hypotension emergent alerts included symptoms associated with systolic blood pressure <80 mmHg or diastolic <45 mmHg. Bradycardia alerts were triggered for symptoms associated with measured heart rate <40 beats per minute. If patients measured a heart rate >140 beats per minute with symptoms, a tachycardia alert was triggered.

At the end of the study, participants were invited to participate in a focus group conducted via Zoom. The purpose of the study was to understand first-hand experiences regarding use of digital health tools for the management of HFrEF.

All patients provided written informed consent to participate in the titration study and group-based interviews following study completion. This study was approved by the Duke University Institutional Review Board.

### 2.4. Outcomes

The purpose of this study was to assess the feasibility of a digital health tool to augment GDMT for patients with HFrEF, and to qualitatively understand patient experiences regarding the use of digital-health tools. At the end of the study period, participants were invited to participate in a remote focus group about their experience in the study. Focus group discussion questions were aimed at understanding experiences related to use of the blood pressure cuff, perception of the mobile interface including receiving texts and completing surveys, interacting with the Story Health coach, and perceived benefits/concerns of the study [18,19]. The session was designed to take approximately one hour, and was held at a time that was convenient for participants. Participants were told at the start that all audio from the discussion would be recorded and transcribed for analysis. To obtain this, we asked questions about use of the remote platform, questions about data confidentiality, and questions on how use of the program impacted the participants’ understanding of heart failure and engagement with health care overall.

Quantitative outcomes included the total number of GDMT initiations, up-titrations, and down-titrations. We also recorded the number of hyperkalemia events attributed to GDMT use requiring GDMT discontinuation, as well as the number of unplanned hospital admissions for acute kidney injury or intravascular volume depletion related to GDMT dose escalation.

### 2.5. Statistical Analyses

Baseline characteristics are reported are median with interquartile ranges. Due to the exploratory nature of this study, no statistical comparisons were planned or performed.

## 3. Results

**Figure 2:**
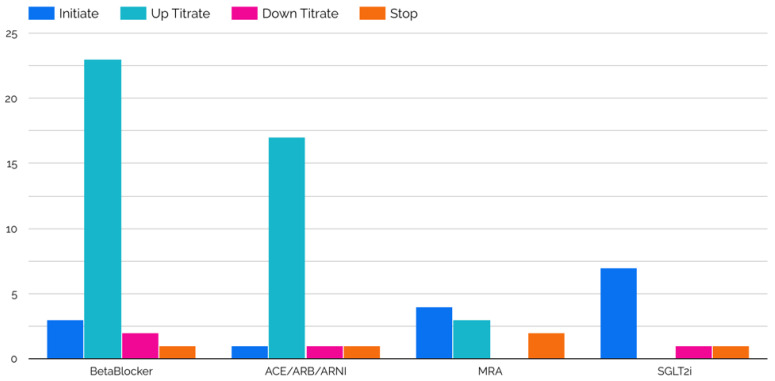
Medication titrations during study period. Legend: ACEi, angiotensin-converting enzyme inhibitor; ARB, angiotensin receptor blocker; ARNI, angiotensin receptor neprilysin inhibitor; MRA, mineralocorticoid receptor antagonist; SGLT2i, sodium glucose transporter-2 inhibitor.

**Figure 3:**
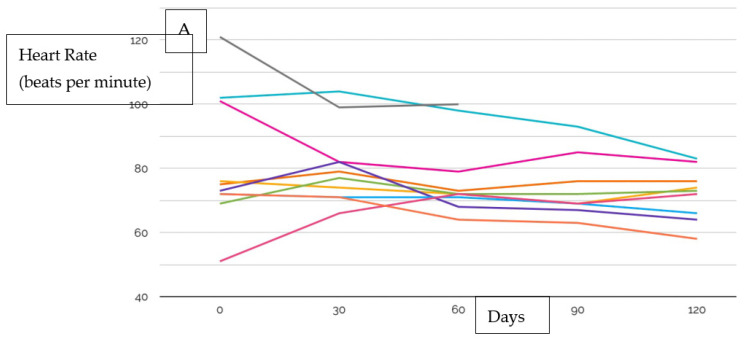

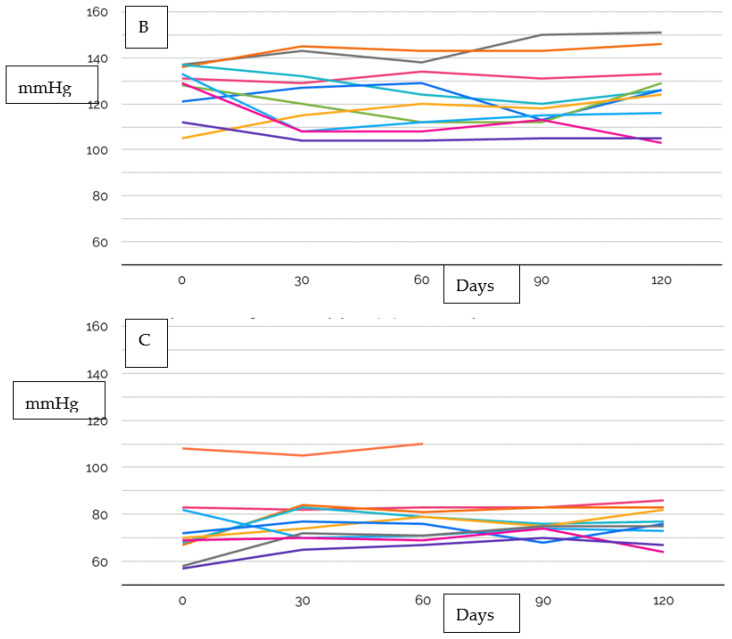
(**A**) Heart rate at beginning and end of the study period plotted for each patient; (**B**) systolic blood pressure at beginning and end of the study period plotted for each patient; (**C**) diastolic blood pressure at beginning and end of the study period plotted for each patient.

Twelve patients were enrolled in the VITAL-HF pilot, with a median follow up of 150 days (IQR, 133–163). No patients that were approached declined to participate. The median age and left ventricular ejection fraction were 52.5 years (IQR, 46.5–63.5) and 25% (IQR, 22.5–35.5), respectively. At baseline, the median heart rate was 72.5 beats per minute (IQR, 67–82.3), median systolic blood pressure was 131 mmHg (IQR, 118.8–136.3), and median diastolic blood pressure was 69.5 mmHg (IQR, 64.8–74.1) (Table 1). During the study period there were a total of 10 GDMT initiations, which included one angiotensin-converting enzyme inhibitor/angiotensin receptor blocker (ACEi/ARB) initiation, two angiotensin receptor neprilysin inhibitor (ARNI) initiations, one beta-blocker initiation, one mineralocorticoid receptor antagonist (MRA) initiation, and four sodium glucose transporter-2 inhibitor (SGLT2i) initiations. There were 52 total GDMT up-titrations, including 7 ACEi/ARB up-titrations, 12 ARNI up-titrations, 24 beta-blocker up-titrations, and 3 MRA titrations. There were 13 down-titrations, which included three ACEi/ARB down-titrations, one ARNI down-titration, five beta-blocker down-titrations, and no MRA down-titrations (Figure 2). Changes in heart rate, as well as systolic and diastolic BP, for each patient are shown in Figure 3. During the study period, four patients required a total of four unplanned emergency room visits and unplanned hospitalizations. Of these, two emergency visits were for cardiac reasons (one was not), and the one hospitalization was not HF-related. There were a total of 45 serum laboratory events during VITAL-HF, and there were two hyperkalemia events requiring de-escalation, direct treatment, discontinuation of therapy, or hospitalization. One patient was enrolled at targeted doses of carvedilol, losartan, spironolactone and, dapagliflozin. Long-acting nitroglycerin and hydralazine were initiated but not tolerated; as such, she was considered to have completed participation of the protocol as there was no further possible treatment. 

### Participant Experiences

Among the participants (n = 5) in the focus group, a majority were female (n = 3) and White (African American n = 2; Hispanic/Latino n = 1). There were four themes that were discussed: (1) regular oversight by a healthcare team, (2) monitoring outside the clinic, (3) frequent medication changes, and (4) concerns about information security (Table 2). 

Participants in the focus group expressed comfort in knowing that there was a team, which included their doctor, who was regularly reviewing their cuff transmissions and symptoms. This extension and access to care on a regular cadence alleviated some of participants’ concerns of uncertainty in daily living, led to an increased feeling of security, and empowered participants to understand decision-making regarding medication changes. The frequency of the medication changes was a concern, including the financial implications of frequent medication changes, as well as individual impact. Finally, of the participants who expressed concerns with data confidentiality and data privacy using a mobile device, the potential benefit of being involved outweighed their concern.

## 4. Discussion

This single-arm feasibility study demonstrates the ability of an automated, algorithm-driven protocol to augment GDMT for patients with HFrEF. This study demonstrates that use of an automated algorithm to titrate GDMT is a potential solution to a commonly identified barrier of clinical inertia when caring for patients with HFrEF [3,7]. Furthermore, this study is unique in several ways, and it begins to address previously identified barriers to GDMT optimization. First, participation in this study, including GDMT titration, was performed remotely. Second, plans for GDMT titration were individualized to each patient. Finally, patients were directly engaged to understand their experiences and feedback.

It is important to note the significant differences between the VITAL-HF Pilot versus other recently reported digital health interventions for patients with HFrEF. The VITAL-HF Pilot was designed with an intervention to augment GDMT for patients with HFrEF. Furthermore, the VITAL-HF Pilot study did not require use of a web-based application on a smart phone, tablet, or personal computer. Recent studies including “The Mobile Application for Self-Care Support of Patients with Chronic Heart Failure” (AppCare-HF) study [20] employed application-based software to assist with HFrEF self-care. Medication adherence was not specifically measured, and there was no part of the study designed to augment GDMT. The “Specialized Primary and Networked Care in Heart Failure (SPAN-CHF) III” trial also required patient interaction with application-based software on a study-provided tablet device. SPAN-CHF III was also not designed to augment GDMT, but rather provide more intensive monitoring and hospitalization prevention [21]. Participants in the VITAL-HF Pilot were provided a blood pressure cuff that automatically transmits data, without patients having to manipulate software or manually enter data.

Recent studies that are similar in design include the “Safety, tolerability, and efficacy of up-titration of guideline-directed medical therapies for acute heart failure” (STRONG-HF) [22]. STRONG-HF randomized patients admitted with acute heart failure to either a highly structured protocol of GDMT up titration or usual care. Whether this strategy can be reproduced in North America, and if it can remain effective, despite a growing population of patients with HFrEF and subsequent increased healthcare resource demands, is not yet known. It is also unknown whether the intervention used in STRONG-HF can be utilized in patients who live in areas that do not allow for recurrent healthcare facility visits for repeated assessment. The VITAL-HF pilot differs in that GDMT titration was performed entirely remotely, without additional need for clinical visits. This strategy may be a more effective and efficient way to manage large populations of patients and allow for care to be delivered to patients who cannot present for frequent assessments. Even more like the VITAL-HF Pilot, Desai et al. report a navigator-driven protocol to augment GDMT for patients with HFrEF, which led to an increase in use of some but not all classes of GDMT [23]. Similar to STRONG-HF, it is unknown whether interventions requiring direct patient interaction can be scaled as the prevalence of HFrEF continues to increase. 

Importantly, as part of the the VITAL-HF Pilot, there were no study-related clinical visits required for study participation outside of routine clinical care. This design feature may allow for greater clinical reach by allowing healthcare team members to manage more patients at further distances from the hospital/clinic, or patients who cannot reliably present for serial in-person assessments. Whether this strategy translates to more clinical efficiency needs to be further studied.

Finally, the VITAL-HF Pilot study further distinguishes itself by having directly engaged participants for feedback following study completion. These unique insights from participants should be considered for future study designs and can be incorporated into daily practice when clinicians are treating patients with HFrEF.

## 5. Limitations

This study involved a small number of participants. However, this study was designed as a pilot study, and no randomization or comparisons were planned. Furthermore, the primary outcome was not to understand changes in clinical, functional, or quality of life outcomes. This study did not directly measure patient adherence to GDMT, and it is unknown whether future studies such as this may improve adherence. Importantly, current rates of HFrEF GDMT utilization are insufficient and, therefore, any potential demonstration of increased use and dose represents a potential improvement in care. 

## 6. Conclusions

The findings of this study suggest that remote titration of GDMT for patients with HFrEF can potentially be performed safely. There was a low rate of medication discontinuation, need for treatment or hospitalization related to medication use. The results of this study provide justification for future adequately powered randomized trials.

## Figures and Tables

**Table 1:** Baseline characteristics.

Age, years (IQR)	52.5 (46.5–63.5)
Left Ventricular Ejection Fraction (IQR)	25% (22.5–35.5)
Female	58.3% (7/12)
Black Race	50.0% (6/12)
Systolic Blood Pressure, mmHg	131 (118.8–136.3)
Diastolic Blood Pressure, mmHg	69.5 (64.8–74.1)
Heart Rate	72.5 (67.0–82.3)
Serum Potassium, mEq/L (IQR)	4.2 (4.05–4.5)
Serum Creatinine mg/dL (IQR)	1.3 (1.0–1.6)
Estimated glomerular filtration rate mL/min/1.73m^2^ (IQR)	59 (39.5–79.5)
Baseline medication use	
ACEi/ARB/ARNI	83.3% (10/12)
Beta-blocker	91.7% (11/12)
Mineralocorticoid receptor antagonist	66.7% (8/12)
SGLT2i	50.0% (6/12)
Ivabradine	0% (0/12)
Hydralazine	16.7% (2/12)
Isosorbide dinitrate	16.7% (2/12)

Continuous variables presented as median (IQR; interquartile range). Legend: ACEi, angiotensin-converting enzyme inhibitor; ARB, angiotensin receptor blocker; ARNI, angiotensin receptor neprilysin inhibitor; SGLT2i, sodium glucose transporter-2 inhibitor.

**Table 2:** Perspectives from VITAL-HF Pilot participants.

Domain/Themes	Examples
Regular oversight by a cohesive team	“I did feel better knowing that somebody was paying attention to and you know monitoring it did make me feel much safer” (Participant 1) “…I love that I was able to check my blood pressure at any time and knowing that somebody was on the other end if something went wrong…” (Participant 2) “Make sure that everything is looking right it was just a little feedback for me to know that yeah I’m on the right track and you know it also helped me remember to take my medication” (Participant 6)
Monitoring outside of the clinic	“it definitely makes you remember to take your medication and remember to take your blood pressure because somebody watching” (Participant 2) “my blood pressure would fluctuate quite a bit and I just went ahead and took the reading when I wasn’t feeling normal so that it would have a a document in there to say hey this is what my blood pressure was doing this time throughout the day” (Participant 6)
Frequent medication changes	“frustrating I had just filled my prescription and in my medication got changed so paying for it twice that’s all” (Participant 2) “…what I didn’t like was that my medications were changed so much that I would go from I feel OK and then all of a sudden medications get changed and my body has to get used to them and then I didn’t feel the greatest for a few days until my body got accustomed to the medications and once my body got accustomed to the medications and I was OK… (Participant 3)
Concerns about Information security	“I’ve been living with the thought you can die in your sleep in this and having to deal with that for over 20 years add take day by day and anybody that wants to know I’m glad to help and when this opportunity to do the research I was willing information more information where people get to know what’s going on with their heart” (Participant 1) “I was a little concerned ‘cause I know that you know text messages not secure at all but you know it’s it’s one of those things that’s helping me so I gave up a little bit of the privacy aspect of it to you know help myself to get better” (Participant 6)

## Data Availability

Because of the sensitive nature of the data collected for this study, requests to access the data set from qualified researchers trained in human subject confidentiality protocols may be sent to Story Health.
